# Entwicklung von Qualitätsstandards für Patient*innen mit axialer Spondyloarthritis zum Einsatz in Deutschland

**DOI:** 10.1007/s00393-021-01019-x

**Published:** 2021-08-11

**Authors:** U. Kiltz, V. Buschhorn-Milberger, K. Albrecht, H.-J. Lakomek, H.-M. Lorenz, M. Rudwaleit, M. Schneider, H. Schulze-Koops, X. Baraliakos, F. Behrens, J. Brandt-Jürgens, H. Haibel, L. Hammel, K. Karberg, H. Kellner, D. Krause, U. Lange, E. Märker-Herrmann, D. Poddubnyy, J. Sieper, U. Syrbe, J. Braun

**Affiliations:** 1 Rheumazentrum Ruhrgebiet, Herne, und Ruhr-Universität Bochum, Claudiusstr. 45, 44649 Herne, Deutschland; 2 Programmbereich Epidemiologie, Deutsches Rheuma-Forschungszentrum (DRFZ), Berlin, Deutschland; 3 Universitätsklinik für Geriatrie, Johannes-Wesling-Klinikum Minden, Minden, Deutschland; 4 Sektion für Rheumatologie, Medizinische Klinik V, Universitätsklinikum Heidelberg, Universität Heidelberg, Heidelberg, Deutschland; 5 Klinik für Innere Medizin und Rheumatologie, Klinikum Bielefeld Rosenhöhe, Bielefeld, Deutschland; 6 Poliklinik, Funktionsbereich und Hiller Forschungszentrum für Rheumatologie, Universitätsklinikum Düsseldorf, Heinrich-Heine-Universität Düsseldorf, Düsseldorf, Deutschland; 7 Sektion Rheumatologie und Klinische Immunologie, Medizinische Klinik und Poliklinik IV, LMU-Klinikum München, Ludwig-Maximilians-Universität München, München, Deutschland; 8 Abteilung Rheumatologie, Universitätsklinikum Frankfurt/Main, Goethe-Universität Frankfurt/Main, Frankfurt/Main, Deutschland; 9 Rheumatologische Schwerpunktpraxis, Ärztehaus am Walter-Schreiber-Platz, Berlin, Deutschland; 10 Medizinische Klinik für Gastroenterologie, Infektiologie und Rheumatologie, Charité – Universitätsmedizin Berlin, Berlin University Alliance, Berlin, Deutschland; 11 DVMB – Deutsche Vereinigung Morbus Bechterew e. V., Schweinfurt, Deutschland; 12 Rheumapraxis Steglitz, Berlin, Deutschland; 13 Schwerpunktpraxis für Rheumatologie und Gastroenterologie, München, Deutschland; 14 Abteilung Rheumatologie, Krankenhaus Neuwittelsbach, München, Deutschland; 15 Rheumatologische Gemeinschaftspraxis, Gladbeck, Deutschland; 16 Abteilung für Rheumatologie und Klinische Immunologie, Kerckhoff Klinik, Justus-Liebig-Universität Gießen, Bad Nauheim, Deutschland; 17 Abteilung für Innere Medizin IV: Rheumatologie und Nephrologie, HELIOS Dr. Horst Schmidt Kliniken Wiesbaden, Wiesbaden, Deutschland; 18 Rheuma Praxis Berlin, Berlin, Deutschland

**Keywords:** Axiale Spondyloarthritis, Ankylosierende Spondylitis, Versorgungsqualität, Qualitätsstandards, Versorgungslücken, Axial spondyloarthritis, Ankylosing spondylitis, Quality of care, Quality standards, Care gaps

## Abstract

Qualitätsstandards (QS) sind messbare Konstrukte, die helfen sollen, Versorgungslücken quantitativ zu erfassen, um langfristig die Versorgungsqualität zu verbessern. Die Assessment of SpondyloArthritis International Society (ASAS) hat kürzlich erstmals internationale QS für das Management von Patient*innen mit axialer Spondyloarthritis (axSpA) konsentiert und veröffentlicht. Die Deutsche Gesellschaft für Rheumatologie (DGRh) hat daraufhin beschlossen, diese Standards durch eine Gruppe von Expert*innen aus unterschiedlichen Versorgungsbereichen zu übersetzen, zu prüfen und ggf. zu übernehmen. Vor diesem Hintergrund wurden erstmals nationale QS für das Management von Patient*innen mit axSpA für Deutschland entwickelt. Hierbei wurde v. a. auf Machbarkeit und Praxisrelevanz geachtet. Letztlich wurden 9 QS definiert, mit denen die Qualität der Versorgung in Deutschland gemessen und verbessert werden kann bzw. soll.

## Abstract (en)

Quality standards (QS) are measurable constructs designed to quantify gaps in care and subsequently to improve quality of care. The Assessment of SpondyloArthritis International Society (ASAS) recently generated and published international QS for the management of patients with axial spondyloarthritis (axSpA) for the first time. The German Society of Rheumatology (DGRh) then decided to translate, review and possibly adopt these standards by a group of experts from different care settings. Against this background, national QS for the management of patients with axSpA for Germany were developed for the first time. The main focus was on feasibility and practical relevance. Ultimately, nine QS were defined with which the quality of care in Germany can and should be measured and improved.

Die axiale Spondyloarthritis (axSpA) ist eine meist chronisch verlaufende entzündlich rheumatische Erkrankung des Achsenskeletts, die oft in den Sakroiliakalgelenken beginnt. Im Verlauf kommt es bei vielen Patient*innen zu strukturellen knöchernen Veränderungen in der Wirbelsäule, initial in Form von Syndesmophyten. Auch periphere Gelenke und Enthesen können in unterschiedlichem Ausmaß betroffen sein, extramuskuloskeletale Manifestationen an Augen, Haut und Darm sind häufig [1, 2].

National und international gibt es zum Teil erhebliche Unterschiede in der Qualität der Gesundheitsversorgung von Patient*innen mit entzündlich rheumatischen Erkrankungen [3, 4]. Entsprechend wurden Defizite in der Versorgung von axSpA-Patient*innen mit biologischen „disease-modifying anti-rheumatic drugs“ (bDMARDs), aber auch im Hinblick auf eine frühe Diagnosestellung nachgewiesen [3, 5, 6].

Die Entwicklung und Festlegung von einfach messbaren Qualitätsstandards (QS) kann helfen, Lücken in der Versorgung zu schließen und die Versorgung zu verbessern [7]. Die Entwicklung von nationalen und internationalen Leitlinien (LL) und Empfehlungen gilt bereits als großer Schritt in Richtung einer soliden Evidenzbasis für therapeutische Entscheidungen in der Medizin allgemein, aber auch in der Rheumatologie im Besonderen [8]. Die Gemeinsamkeit von LL und QS besteht darin, dass beide durch Expert*innen in meist aufwendigen Prozessen entwickelt werden. Der Unterschied zwischen LL und QS dagegen ist, dass Erstere evidenzbasierte Empfehlungen für das Management von Erkrankungen, z. T. auch in unterschiedlichen Stadien, hervorbringen, während Letztere sich durch den Einsatz von messbaren Qualitätskonstrukten auszeichnen, indem Instrumente definiert werden, mit denen Versorgungsqualität in den Domänen Strukturqualität, Prozessqualität und Ergebnisqualität gemessen werden kann [9].

Für einige Erkrankungen aus dem rheumatologischen Erkrankungskreis, wie z. B. der rheumatoiden Arthritis, sind international bereits Instrumente zur Qualitätsbeurteilung vorgeschlagen worden [10–13]; für axSpA war das bisher nicht der Fall. Die Assessment of Spondyloarthritis International Society (ASAS) hatte es sich daher zur Aufgabe gemacht, einfach einsetzbare international akzeptierte QS für Patient*innen mit axSpA zu entwickeln. Dabei orientierte sich die Organisation an der Vorgehensweise des vom National Institute for Health and Care Excellence (NICE) entwickelten Ansatz zur Entwicklung von QS [14, 15]. Dieser zeichnete sich durch die Beteiligung aller relevanten Interessengruppen unter Einbeziehung der vorliegenden wissenschaftlichen Evidenz für die Entwicklung von QS aus.

Grundsätzlich bestehen QS aus 2 Komponenten: einer Qualitätsaussage und einer Qualitätsmessung. Die Qualitätsaussage, auf die sich eine Expert*innenkommission geeinigt hat, definiert die gewünschte Versorgungsqualität auf Basis einer prägnanten und potenziell messbaren Aussage, die mit vorhandenen evidenzbasierten Leitlinien kompatibel ist bzw. sein sollte. Hierbei geht es darum, einen Versorgungsbereich zu identifizieren, der sowohl relevant als auch erreichbar und messbar ist, um auf dieser Basis die gewünschte Versorgungsqualität zu definieren. Jeder Aussage wird eine Rationale zugrunde gelegt, die auf wissenschaftlicher Evidenz, verfügbaren LL, bestehenden Empfehlungen und der Einschätzung der Expert*innen auf mögliche Auswirkungen der QS auf Zielgruppen (Dienstleister, öffentliches Gesundheitswesen und Soziales, Patient*innen etc.) beruht. Ob die in der Qualitätsaussage gewünschte Versorgungsqualität erreicht wurde, kann durch Qualitätsmessinstrumente quantifiziert werden. Die dafür erforderlichen Instrumente sind für die etablierten Qualitätsdomänen Struktur‑, Prozess- und Ergebnisqualität unterschiedlich. Um ein besseres Ergebnis, d. h. eine bessere Versorgungsqualität zu erreichen, sind eine solide Struktur und gut durchdachte Prozesse erforderlich. Für die Instrumente zur Messung der Qualität muss jeweils definiert werden, welche Größe im Zähler und welche im Nenner steht [15–17].

Im Auftrag der Deutschen Gesellschaft für Rheumatologie (DGRh) und in Anlehnung an die von der ASAS publizierten Vorgabe sowie auf Basis der deutschen S3-Leitlinie war das Ziel dieses Projektes, die international entwickelten QS an nationale Gegebenheiten anzupassen und QS für axSpA-Patient*innen in Deutschland zu entwickeln.

## Methoden

Zu Beginn des Projektes im September 2019 wurden eine Steuerungsgruppe (KA, JB, UK, H‑JL, H‑ML, MR, MS, HS-K) und eine Arbeitsgruppe (AG) mit ausgewählten Expert*innen gebildet. Die Steuerungsgruppe entschied initial über die zu verwendende Methodik sowie die Beteiligung verschiedener Interessengruppen. Die AG setzte sich aus Mitgliedern der Steuerungsgruppe sowie verschiedenen Interessengruppen (stationär und ambulant tätige Rheumatolog*innen und einem Patientenvertreter der Deutschen Vereinigung Morbus Bechterew e. V. [DVMB]) zusammen.

Die internationalen von ASAS publizierten QS für axSpA wurden als Grundlage für die Entwicklung akzeptiert und die Adaptation für den deutschsprachigen Raum geplant, wobei angesichts der aktuellen Pandemie keine Expert*innen aus Österreich bzw. der Schweiz hinzugezogen wurden.

Die geplante Methodik sah zunächst die Übersetzung der englischen Originalfassung in eine deutsche Version durch Mitglieder der AG auf Grundlage eines Vorschlags von JB und UK vor. Im Anschluss daran hatten die Mitglieder der AG die Möglichkeit, diesen Übersetzungsvorschlag zu kommentieren. In einem virtuellen Treffen wurden dann Änderungswünsche diskutiert, und anschließend wurde die finale Übersetzung konsentiert. Vor dem Hintergrund der methodischen Vorgaben führten die Mitglieder der AG eine inhaltliche Prüfung der Empfehlungen durch, passten diese an die deutsche Versorgungssituation an und identifizierten mögliche Hinderungsfaktoren für die Verbreitung und Umsetzung der QS in Deutschland. Abschließend wurden alle Mitglieder der AG gebeten, zu jedem einzelnen QS ihre Zustimmung oder Ablehnung zu bekunden sowie den Grad ihrer Zustimmung mithilfe einer numerischen Rating-Skala (NRS) mitzuteilen (Grad 0 = stimme nicht zu, Grad 10 = stimme voll zu).

## Ergebnisse

Der erste Übersetzungsvorschlag (JB) wurde im Sommer 2020 in einem ersten Umlaufverfahren diskutiert und in einem wegen der Corona-Pandemie virtuell durchgeführten Treffen im September 2020 erneut kommentiert und letztlich konsentiert. Im Rahmen des Abstimmungsprozesses wurde zunächst die englische Version kritisch geprüft und befunden, dass diese nicht 1:1 auf Deutschland übertragen werden kann. Entsprechend wurden einige Formulierungen und hier insbesondere der QS 5 abgeändert und neu formuliert. Dieser QS weicht nun deutlich vom englischsprachigen Original der ASAS ab.

Insgesamt nahmen 15 AG-Mitglieder an der Abstimmung der QS während des virtuellen Meetings teil. An der anschließend durchgeführten Online-Umfrage nahmen 10 der 15 eingeladenen Mitglieder teil und bewerteten den Grad der Zustimmung (NRS 0–10) jedes einzelnen Qualitätsstandards (Tab. 1). Hierbei wurde ein minimaler Grad der Zustimmung bei QS 8 (Kurzfristiger Termin) mit 7,8 ± 2,4 und ein maximaler Grad der Zustimmung bei QS 5 (Leitliniengerechte Therapieeskalation) mit 9,8 ± 0,4 erreicht. Im Durchschnitt lag der Grad der Zustimmung aller QS bei 9,3 ± 0,7.

**Table 1:** 

Qualitätsstandard	Thema	Zustimmung ja/nein	Grad der Zustimmung (NRS 0–10), MW ± SD
QS 1	Überweisung	15/0	8,9 ± 1,6
QS 2	Zeit bis zum Termin	15/0	9,6 ± 0,7
QS 3	Diagnostische Abklärung	15/0	9,7 ± 0,5
QS 4	Monitoring	15/0	9,7 ± 0,5
QS 5	Leitliniengerechte Therapieeskalation	13/0	9,8 ± 0,4
QS 6	Nicht-pharmakologische Behandlung	15/0	9,7 ± 0,6
QS 7	Patient*innenschulung	15/0	8,8 ± 1,5
QS 8	Kurzfristiger Termin	15/0	7,8 ± 2,4
QS 9	Jährliche Beurteilung	15/0	9,1 ± 1,3

### Übersetzung der einzelnen QS

Es wurden insgesamt 9 QS der internationalen Version übersetzt. Vor der Abstimmung der einzelnen QS diskutierten die AG-Teilnehmer*innen den Gebrauch einer geschlechtsneutralen Sprache. Im letztlich durchgeführten Abstimmungsprozess stimmten 14 von 15 Mitgliedern für die Verwendung einer geschlechtsneutralen Sprache in Form eines Gendersternchens (1 Mitglied enthielt sich). Kritisch angemerkt wurde, dass sich die Lesbarkeit des Manuskripts durch die Gendersternchen leider deutlich erschwert.

Im Folgenden wird auf die einzelnen QS eingegangen, und die Diskussionen und Kommentare während der Abstimmung werden beschrieben und erläutert.

Die Ausformulierung der QS mit Rationale und Messwerten zeigen Tab. 2, 3, 4, 5, 6, 7, 8, 9 und 10.

**Table 2:** 

Domäne	Standard	Rationale	Qualitätsmessung, Kategorie Struktur	Qualitätsmessung, Kategorie Prozess, Zähler	Qualitätsmessung, Kategorie Prozess, Nenner
Überweisung	Patient*innen mit Verdacht auf axSpA werden innerhalb von 3 Werktagen zur diagnostischen Abklärung an eine*n Rheumatolog*in überwiesen	Eine axSpA wird von Nicht-Rheumatolog*innen häufig nicht erkannt, was zu erheblichen Verzögerungen bei Diagnose und Behandlung führt. Es gibt keinen diagnostischen Test, der allein eine ausreichende Sensitivität oder Spezifität für die Diagnose axSpA aufweist. Die ASAS-Empfehlungen enthalten Kriterien für die Entscheidung zu einer zeitnahen Überweisung von Patient*innen mit Verdacht auf axSpA in die Rheumatologie. Die auf Expert*innenmeinung basierende Wahl des Zeitraums von 3 Arbeitstagen soll eine sofortige Überweisung (z. B. durch Hausärzt*innen) bewirken	Vorhaltung von Maßnahmen und Strukturen (einschließlich Vorkehrungen zur Sensibilisierung für Anzeichen und Symptome von axSpA) und vereinbarte Überweisungsverfahren, um sicherzustellen, dass Patient*innen mit Verdacht auf axSpA innerhalb von 3 Werktagen an eine*n Rheumatolog*in überwiesen werden	Die Anzahl der Patient*innen mit Verdacht auf axSpA, die innerhalb von 3 Werktagen an Rheumatolog*innen überwiesen wurden. Die 3 Tage werden zum einen an der Dokumentation der Verdachtsdiagnose und zum anderen an der Ausstellung des Überweisungsscheins gemessen	Die Anzahl der Patient*innen mit Verdacht auf axSpA

**Table 3:** 

Domäne	Standard	Rationale	Qualitätsmessung, Kategorie Struktur	Qualitätsmessung, Kategorie Prozess, Zähler	Qualitätsmessung, Kategorie Prozess, Nenner
Zeit bis zum Termin bei Rheumatolog*innen	Patient*innen mit Verdacht auf axSpA werden innerhalb von 3 Wochen nach der Überweisung von einem*einer Rheumatolog*in untersucht	Eine schnelle Überweisung von Patient*innen mit Verdacht auf axSpA ist wichtig, um Verzögerungen bei der Diagnosestellung zu vermeiden und die Wahrscheinlichkeit eines frühen Behandlungsbeginns zu erhöhen. Ein*e Rheumatolog*in (der Terminus bezieht sich auf das gesamte rheumatologische Team, bestehend aus Ärzt*innen, Pflegepersonal und anderen medizinischen Fachkräften) ist in der Lage, axiale und periphere Manifestationen sowie extraartikuläre Manifestationen und Komorbiditäten zu identifizieren. Angesichts der potenziell nachteiligen Auswirkungen einer verspäteten Diagnose benötigen Patient*innen mit diesen Symptomen und Anzeichen einen ersten Termin innerhalb von 3 Wochen. Der Zeitrahmen basiert auf Expert*innenmeinung, um die Vergabe von zeitnahen Terminen anzuregen. Die Frist von 3 Wochen bezieht sich auf den ersten Termin. Zusätzliche Untersuchungen, die für die Diagnosefindung erforderlich sind, können auch nach dem ersten Termin erfolgen	Vorhaltung von Maßnahmen und Strukturen, einschließlich einer ausreichenden Anzahl von Rheumatolog*innen, um sicherzustellen, dass Patient*innen mit Verdacht auf axSpA innerhalb von 3 Wochen nach der Überweisung von einem*einer Rheumatolog*in gesehen werden	Die Anzahl der Patient*innen mit Verdacht auf axSpA, die von einem*einer Rheumatolog*in innerhalb von 3 Wochen nach der Überweisung untersucht werden	Die Anzahl der Patient*innen mit Verdacht auf axSpA, die an eine*n Rheumatolog*in überwiesen wurden

**Table 4:** 

Domäne	Standard	Rationale	Qualitätsmessung, Kategorie Struktur	Qualitätsmessung, Kategorie Prozess, Zähler	Qualitätsmessung, Kategorie Prozess, Nenner
Diagnostische Abklärung durch Rheumatolog*innen	Bei Verdacht auf axSpA wird die rheumatologische Abklärung innerhalb von 2 Monaten abgeschlossen	Eine rechtzeitige Abklärung durch eine*n Rheumatolog*in ist notwendig, um eine korrekte Diagnose stellen zu können. Durch eine frühzeitige Diagnose wird der langfristige Krankheitsverlauf verbessert und die Lebensqualität gesteigert. Die diagnostische Abklärung beinhaltet die Identifizierung von SpA-Symptomen, Laborparameter und eine entsprechende Bildgebung. Diese sollte innerhalb von 2 Monaten nach der ersten Vorstellung bei dem*der Rheumatolog*in abgeschlossen sein	Vorhaltung von Maßnahmen und Strukturen, einschließlich einer ausreichenden Anzahl von Rheumatolog*innen und Einrichtungen sowie der Zugang zu diesen Einrichtungen innerhalb des vorgegebenen Zeitrahmens, um sicherzustellen, dass Patient*innen mit Verdacht auf axSpA innerhalb von 2 Monaten nach der ersten Vorstellung durch eine*n Rheumatolog*in eine diagnostische Abklärung erhalten	Die Anzahl der Patient*innen mit Verdacht auf axSpA, bei denen eine diagnostische Abklärung innerhalb von 2 Monaten nach der ersten Vorstellung abgeschlossen wurde	Die Anzahl der Patient*innen mit Verdacht auf axSpA, die der*die Rheumatolog*in vor mehr als 2 Monaten erstmalig gesehen hat

**Table 5:** 

Domäne	Standard	Rationale	Qualitätsmessung, Kategorie Struktur	Qualitätsmessung, Kategorie Prozess, Zähler	Qualitätsmessung, Kategorie Prozess, Nenner
Monitoring	Die Krankheitsaktivität von Patient*innen mit axSpA wird unter Supervision durch Rheumatolog*innen mit validierten Messinstrumenten mindestens alle 6 Monate evaluiert	Die Beurteilung der Krankheitsaktivität ist wegen des Zusammenhangs zwischen klinischer Krankheitsaktivität und Syndesmophytenbildung sowie zwischen Krankheitsaktivität, Funktionsfähigkeit und gesundheitsbezogener Lebensqualität von Bedeutung. Die Überwachung der Krankheitsaktivität durch eine*n Rheumatolog*in (der Terminus bezieht sich auf das gesamte rheumatologische Team, bestehend aus Ärzt*innen, Pflegepersonal, rheumatologischer Fachassistenz und anderen medizinischen Fachkräften) ist wegen der vielfältigen und mehrdeutigen klinischen Symptome der Krankheitsaktivität wie Schmerzen und Behinderungen erforderlich. Eine Bewertung der Krankheitsaktivität mittels ASDAS wird empfohlen. Durch den regelmäßigen Einsatz der Messinstrumente wird sichergestellt, dass die Behandlung von Patient*innen mit axSpA bei Bedarf angepasst werden kann	Vorhaltung von Maßnahmen und Strukturen, um sicherzustellen, dass Patient*innen mit axSpA mindestens alle 6 Monate mit validierten Messinstrumenten evaluiert werden	Die Anzahl der Patient*innen, bei denen vor mehr als 6 Monaten eine axSpA diagnostiziert wurde und bei denen die Krankheitsaktivität mindestens alle 6 Monate mit validierten Messinstrumenten überwacht wurde	Die Anzahl der Patient*innen, bei denen vor mehr als 6 Monaten eine axSpA diagnostiziert wurde

**Table 6:** 

Domäne	Standard	Rationale	Qualitätsmessung, Kategorie Struktur	Qualitätsmessung, Kategorie Prozess, Zähler	Qualitätsmessung, Kategorie Prozess, Nenner
Leitliniengerechte Therapieeskalation	Bei Patient*innen mit axSpA und hoher Krankheitsaktivität ist das zentrale Ziel, eine Remission durch Therapieeskalation entsprechend der aktuellen S3-Leitlinie zu erreichen	Eine Therapieeskalation ist wichtig, um die Krankheitsaktivität zu kontrollieren. Dies erfolgt mit dem Ziel, idealerweise eine Remission oder einen Zustand niedriger Krankheitsaktivität zu erreichen, um den Einfluss der Erkrankung auf die Funktionsfähigkeit und den Alltag der Patient*innen zu minimieren. Patient*innen, die trotz konventioneller Therapie eine hohe Krankheitsaktivität haben, sollten mit ihrem*ihrer Rheumatolog*in die verschiedenen Therapiemöglichkeiten besprechen. Die aktuelle S3-Leitlinie für das Management von Patient*innen mit axSpA enthält Kriterien für den Einsatz von medikamentösen und nichtmedikamentösen Therapien bei Patient*innen mit hoher Krankheitsaktivität. Die Therapieentscheidung sollte auf einer gemeinsamen Entscheidung zwischen Patient*innen und Rheumatolog*innen beruhen	Vorhaltung von Maßnahmen und Strukturen, um sicherzustellen, dass Patient*innen mit axSpA und hoher Krankheitsaktivität gemäß der aktuellen S3-Leitlinie behandelt werden können, um die Chance auf Remission oder geringe Krankheitsaktivität zu maximieren	Die Anzahl der Patient*innen mit axSpA und hoher Krankheitsaktivität, bei denen eine Therapieeskalation gemäß der aktuellen S3-Leitlinie durchgeführt wurde	Die Anzahl der Patient*innen mit axSpA und hoher Krankheitsaktivität

**Table 7:** 

Domäne	Standard	Rationale	Qualitätsmessung, Kategorie Struktur	Qualitätsmessung, Kategorie Prozess, Zähler	Qualitätsmessung, Kategorie Prozess, Nenner
Nicht-pharmakologische Behandlung	Patient*innen mit axSpA werden über den Nutzen regelmäßiger Bewegungsübungen informiert	Körperliche Aktivität sollte bei Patient*innen mit axSpA ein integraler Bestandteil der Standardversorgung während des gesamten Krankheitsverlaufs darstellen. Es ist wichtig, dass Patient*innen mit axSpA über den Nutzen regelmäßiger Bewegungsübungen informiert werden, um Schmerzen und Steifigkeit zu reduzieren. Außerdem können hierdurch die kardiorespiratorische Fitness und Belastbarkeit verbessert und damit auch das Risiko für Herz-Kreislauf-Erkrankungen reduziert werden. Vorteile eines regelmäßigen Trainings sollten dabei aktiv angesprochen werden, um die Patient*innen zu unterstützen, ihre Funktionsfähigkeit zu verbessern und ihre Lebensqualität zu erhalten	Vorhaltung von Strukturen und Maßnahmen, um Patient*innen mit axSpA zu ermutigen, regelmäßig Bewegungsübungen durchzuführen	Die Anzahl der Patient*innen mit axSpA, die über den Nutzen regelmäßiger Bewegungsübungen informiert wurden	Die Anzahl der mit axSpA diagnostizierten Patient*innen

**Table 8:** 

Domäne	Standard	Rationale	Qualitätsmessung, Kategorie Struktur	Qualitätsmessung, Kategorie Prozess, Zähler	Qualitätsmessung, Kategorie Prozess, Nenner
Schulung als Basis zum Selbstmanagement	Patient*innen mit axSpA wird innerhalb von 2 Monaten nach Diagnosestellung eine Schulung über die Erkrankung angeboten mit dem Ziel, ein optimales Selbstmanagement zu erreichen	Eine strukturierte Patient*innenschulung ist hilfreich, um das Verständnis und das Selbstmanagement von axSpA zu ermöglichen und das Risiko von Komplikationen zu reduzieren. Es ist wichtig, dass die Patient*innen lernen, wie sie mit ihren Symptomen umgehen, ihre Schmerzen und Sorgen reduzieren und ihre Funktionsfähigkeit und Lebensqualität verbessern können. Schulungsprogramme sollen Informationen über die Krankheit, den diagnostischen Abklärungsprozess, einen gesunden Lebensstil (körperliche Aktivität und Raucherentwöhnung) und Behandlungsmöglichkeiten einschließlich möglicher Nebenwirkungen enthalten. Medizinisches Fachpersonal kann die Fähigkeit der Patient*innen, ihren Zustand selbst zu steuern, unterstützen, indem es ermutigende Hinweise zur Ursache (Entzündung), aber auch beruhigende Hinweise zum Verlauf der Erkrankung (Risiko einer fortschreitenden Behinderung) und zur Bedeutung eines aktiven Lebensstils gibt. Weitere Beratungen sollten sich über den gesamten Krankheitsverlauf der Patient*innen erstrecken	Vorhaltung von Strukturen und Maßnahmen, um sicherzustellen, dass Angehörige der Gesundheitsberufe Zugang zu Informationen und Kenntnissen haben, die erforderlich sind, um den Schulungsbedarf der Patient*innen vollständig zu decken und um deren Fragen beantworten zu können	Die Anzahl der Patient*innen mit einer diagnostizierten axSpA, die innerhalb von 2 Monaten nach Diagnosestellung eine strukturierte Patient*innenschulung erhalten haben	Die Anzahl der mit axSpA diagnostizierten Patient*innen

**Table 9:** 

Domäne	Standard	Rationale	Qualitätsmessung, Kategorie Struktur	Qualitätsmessung, Kategorie Prozess, Zähler	Qualitätsmessung, Kategorie Prozess, Nenner
Kurzfristiger Temin	Patient*innen mit axSpA und einem Schub der Erkrankung oder potenziellen Nebenwirkungen der Medikation werden innerhalb von 2 Werktagen nach Kontaktaufnahme von einem*einer Rheumatolog*in beraten	Patient*innen mit axSpA können komplexe Bedürfnisse haben, da das klinische Bild durch Krankheitsschübe, eine Schmerzintensivierung aufgrund anderer Ursachen oder medikamentenbedingte Nebenwirkungen geprägt sein kann. Der schnelle und unverzügliche Zugang zu einer rheumatologischen Beratung kann die Auswirkungen der Erkrankung auf die Lebensqualität der Person mindern, sodass er*sie seine*ihre Alltagstätigkeiten fortsetzen kann und sich die Wahrscheinlichkeit von Schäden durch die Erkrankung oder durch Nebenwirkungen verringert. Der schnelle Zugang kann über alle möglichen Formen der Kontaktaufnahme mit dem*der Rheumatolog*in (persönlich, telefonisch oder über das Internet) erfolgen (der Terminus bezieht sich auf das gesamte rheumatologische Team, bestehend aus Ärzt*innen, Pflegepersonal, rheumatologischer Fachassistenz und anderen medizinischen Fachkräften)	Vorhaltung von Strukturen und Maßnahmen, um sicherzustellen, dass Patient*innen mit axSpA innerhalb von 2 Werktagen nach Kontaktaufnahme von einem*einer Rheumatolog*in beraten werden	Die Anzahl der Patient*innen mit axSpA, die einen Schub der Erkrankung oder Nebenwirkungen der Medikation haben und innerhalb von 2 Werktagen nach Kontaktaufnahme mit dem*der Rheumatolog*in beraten wurden	Die Anzahl der mit axSpA diagnostizierten Patient*innen, die um einen dringenden Termin gebeten haben

**Table 10:** 

Domäne	Standard	Rationale	Qualitätsmessung, Kategorie Struktur	Qualitätsmessung, Kategorie Prozess, Zähler	Qualitätsjmessung, Kategorie Prozess, Nenner
Jährliche Beurteilung	Patient*innen mit axSpA werden jährlich umfassend von einem*einer Rheumatolog*in beurteilt	Eine jährliche Beurteilung ist wichtig, um sicherzustellen, dass alle Aspekte der Krankheit unter Kontrolle sind. Die regelmäßige Überprüfung bietet die Gelegenheit, den Patient*innen im Hinblick auf das aktuelle Krankheitsmanagement und die Erfordernisse weiterer Unterstützungsmaßnahmen zu beurteilen. Damit soll sichergestellt werden, dass der*die Patient*in seine*ihre Gesundheit, seine*ihre gesellschaftliche Teilhabe und seine*ihre Lebenszufriedenheit verbessern kann. Der Fokus sollte nicht nur auf den klinischen Symptomen und der Schwere der Erkrankung, sondern auch auf Komorbiditäten, insbesondere kardiovaskulären Risikofaktoren und Osteoporose sowie auf Aspekten der Teilhabe liegen. Diese Themen sollten individualisiert angesprochen und können durch das gesamte rheumatologische Team (Ärzt*innen, Pflegepersonal, rheumatologische Fachassistenz und andere medizinische Fachkräfte) adressiert werden. Bei Bedarf wird der*die Patient*in zu anderen Fachärzt*innen überwiesen	Nachweis von Maßnahmen für Patient*innen mit axSpA, bei welchen eine umfassende jährliche Beurteilung durch eine*n Rheumatolog*in koordiniert wird	Die Anzahl der Patient*innen mit axSpA, die vor mehr als 1 Jahr diagnostiziert wurden und deren letzte umfassende Beurteilung innerhalb von 12 Monaten nach Diagnosestellung bzw. der letzten vorgenommenen Beurteilung lag	Die Anzahl der Patient *innen, bei denen die axSpA vor mehr als 1 Jahr diagnostiziert wurde

### QS 1: Überweisung

Zunächst wurde über die Änderung des Begriffes „Überweisung“ in „Zuweisung“ diskutiert, da der Begriff der „Überweisung“ ein kassenärztlicher Terminus ist. Mehrheitlich wurde sich dann jedoch für den Begriff „Überweisung“ entschieden.

Im Unterschied zu den internationalen ASAS-QS wurde bei der deutschen Übersetzung auf den Begriff „lokal“ bei der Kategorie Struktur „Vorhaltung [lokaler] Maßnahmen und Strukturen […]“ verzichtet, da lokal zu eng definiert ist und in einigen Teilen Deutschlands eine überregionale oder sektorale Versorgung stattfindet, welche durch den Begriff „lokal“ nicht abgebildet wird. Ebenfalls angemerkt wurde, dass geeignete Maßnahmen und Strukturen besser grundsätzlich vorhanden und nicht ortsbezogen begrenzt sein sollten (Tab. 2).

Nach erfolgten Änderungen stimmten 15 der 15 Mitglieder für die finale Übersetzung.

### QS 2: Zeit bis zum Termin bei Rheumatolog*innen

Es wurde die Frage diskutiert, was in den internationalen ASAS QS mit „specialist“ gemeint ist. Während in anderen Ländern zum Teil auch in verschiedenen Fachrichtungen tätige Ärzt*innen in der Versorgung von Patient*innen mit rheumatischen Erkrankungen tätig sind, ist in Deutschland vorwiegend der*die internistische Rheumatolog*in, meist in Kooperation mit dem*der Hausarzt*ärztin, für die Versorgung verantwortlich.

Damit wurde von der AG hinsichtlich der ärztlichen Zuständigkeit für das Management von Patient*innen mit axSpA klar definiert, dass bei allen QS grundsätzlich der*die internistische Rheumatolog*in als verantwortliche*r Facharzt*ärztin gemeint ist. Grundlage hierfür sind die für eine adäquate Patient*innenversorgung erforderliche internistische Facharztausbildung mit fundierter Kenntnis über die differenzialdiagnostische Abklärung sowie ein breites Wissen über medikamentöse Therapieprinzipien und deren Management im Langzeitverlauf auch hinsichtlich unerwünschter Wirkungen und Komplikationen.

Daher wurde die Übersetzung in „Zeit […] bei Rheumatolog*innen“ anstatt „Zeit […] bei Spezialist*innen“ abgeändert (Tab. 3).

Nach erfolgten Änderungen stimmten 15 der 15 Mitglieder für die finale Übersetzung.

### QS 3: Diagnostische Abklärung durch Rheumatolog*innen

Die ursprüngliche englische Benennung „assessment“ wurde kritisch diskutiert vor dem Hintergrund, dass die erfolgten Untersuchungen und Evaluierungen vom Kontext abhängen und somit nicht per se ein reines Assessment darstellen. Es wurde entschieden, dass mit dem Begriff „diagnostische Abklärung“ den verschiedenen klinischen, radiologischen und laborchemischen Untersuchungen besser Rechnung getragen wird.

Ebenfalls diskutiert wurde, ob das „langfristige Outcome“ oder der „langfristige Krankheitsverlauf“ betrachtet werden soll. Da das „langfristige Outcome“ im engeren Sinne einen in der Zukunft liegenden Status der Erkrankung und nicht den Verlauf der Krankheit meint, wurde entschieden, den Begriff „Krankheitsverlauf“ zu nehmen (Tab. 4).

Nach erfolgten Änderungen stimmten 15 der 15 Mitglieder für die finale Übersetzung.

### QS 4: Monitoring

Grundsätzlich sollten die mit der Festlegung eines QS beabsichtigten Ziele nicht nur für die Versorgung wünschenswert, sondern auch erreichbar sein. Daher wurde in diesem QS ein Zeitraum von 6 Monaten gewählt. Dies wurde von einigen Mitgliedern allerdings eher als zu lang als zu kurz eingestuft.

In Zukunft wäre die mögliche Delegation einiger ärztlicher Aufgaben z. B. an eine rheumatologische Fachassistenz wünschenswert, daher wurde in diesem QS die entsprechende Formulierung gewählt, dass Patient*innen „[…] unter Supervision einer*s Rheumatolog*in […]“ regelmäßig evaluiert werden, anstatt diesen QS lediglich auf den Tätigkeitsbereich der*s Rheumatolog*in zu begrenzen (Tab. 5).

Nach erfolgten Änderungen stimmten 15 der 15 Mitglieder für die finale Übersetzung.

### QS 5: Leitliniengerechte Therapieeskalation

Generell wurde angemerkt, dass im Gegensatz zur internationalen Situation, die dadurch gekennzeichnet ist, dass in einigen Ländern bei Patient*innen mit axSpA relativ wenig bDMARDs verordnet werden, die Versorgungslücke in Deutschland durch eine unzureichende leitliniengerechte Therapie insofern bedingt ist, als dass es Anhaltspunkte für eine Unterversorgung dieser Patient*innen mit Physiotherapie gibt (hierauf wird im QS 6 näher eingegangen) [18, 19]. Für eine qualitativ mangelhafte Versorgung mit NSAR gibt es insgesamt keine sicheren Hinweise [20]. Auch die Möglichkeit einer medikamentösen Überversorgung wurde thematisiert. Einige AG-Mitglieder bemängelten in diesem Zusammenhang, dass die aktuellen QS die Möglichkeit einer Deeskalation der Therapie nur unzureichend adressieren.

Prinzipiell wurde in diesem Zusammenhang betont, dass eine Verordnung von Medikamenten nicht nur medizinisch, sondern auch im Sinne eines adäquaten wirtschaftlichen Handelns vertretbar sein sollte. Dazu gehört auch, dass die Verordnung eines bDMARDs bei vorliegender hoher Krankheitsaktivität nicht nur zu einer Versorgungsverbesserung für den*die jeweilige*n Patient*in führen kann, sondern durch die Vermeidung einer Hospitalisierung auch durchaus wirtschaftlich sinnvoll ist, weil damit Kosten für stationäre Versorgung und Arbeitsunfähigkeit eingespart werden können [21].

Zudem wurde die Begrifflichkeit „konventionelle Therapie“ diskutiert. Im internationalen rheumatologischen Wortschatz fallen unter den Begriff „conventional synthetic“ (cs)DMARDs gängige Medikamente der Basisversorgung wie MTX, Leflunomid oder Sulfasalazin, für die eine krankheitsmodifizierende Wirkung im Sinne einer Hemmung der Röntgenprogression nachgewiesen wurde. Das gilt allerdings eigentlich nur für die rheumatoide Arthritis, bzw. wurde bei Patient*innen mit dieser Erkrankung gezeigt. Der Terminus wurde für axSpA übernommen, obwohl eine solche Wirkung bei dieser Erkrankung weder nachgewiesen wurde noch anzunehmen ist. Die Biologika laufen unter dem Begriff „biological“ (b)DMARDs, wobei es neuerdings auch „targeted synthetic“ (ts)DMARDs wie die Januskinaseinhibitoren gibt, der erste ist inzwischen zugelassen.

Kritisch wurde bemerkt, dass dieser QS nicht das Problemfeld des Zuganges zur Therapie beleuchtet. Explizit wurden hier Patient*innen ohne Krankenversicherung angeführt, welche zwar zum aktuellen Zeitpunkt in Deutschland ein vergleichsweise kleines Problem darstellen, was jedoch in den nächsten Jahren zunehmend an Bedeutung gewinnen könnte. Das Statistische Bundesamt hat kürzlich Zahlen veröffentlicht, wonach im Jahr 2019 immerhin 143.000 Menschen, also knapp 0,1 % der Bevölkerung, nicht krankenversichert waren. Im Vergleich dazu waren es im Jahr 2015 nur rund 79.000 ([22]; Tab. 6).

Nach erfolgten Änderungen stimmten die 13 noch anwesenden Mitglieder für die finale Übersetzung. Da dieses QS als Letztes abgestimmt wurde, konnten 2 Mitglieder nicht an der Abstimmung teilnehmen.

### QS 6: Nicht-pharmakologische Behandlung

Der Nutzen regelmäßiger Bewegung wurde während der Diskussion weiter spezifiziert, und schlussendlich einigte sich die Gruppe darauf, dass dem*der Patient*in Informationen über die regelmäßige Durchführung von Bewegungsübungen vermittelt werden sollten, da nicht jegliche Art der Bewegung, sondern v. a. gezielte Bewegungsübungen und regelmäßige körperliche Aktivität einen Benefit bei der Therapie der axSpA gezeigt haben ([23]; Tab. 7).

Nach erfolgten Änderungen stimmten 15 von 15 Mitgliedern für die finale Übersetzung.

### QS 7: Patient*innenschulung

Gemeinhin mag eine strukturierte Patient*innenschulung sinnvoll erscheinen und ist auch von Expert*innen als solche angesehen, jedoch ist kritisch anzumerken, dass es bislang nur begrenzt wissenschaftliche Evidenz dafür gibt, eine strukturierte Schulung für das Krankheitsverständnis der Patient*innen als besser einzustufen als eine Informationsvermittlung durch den*die behandelnde*n Arzt*Ärztin [24–27]. Strukturierte Schulungen sollen auch Hilfe zur Selbsthilfe darstellen. Hierbei ist allerdings von erheblichen individuellen Unterschieden hinsichtlich des benötigten Schulungsbedarfes auszugehen. Insofern stellte sich die Frage, ob lediglich die Anzahl strukturierter Patient*innenschulungen bereits ein Qualitätsmaß darstellt oder vielleicht doch eher das Ergebnis, wie z. B. das Wissen und das Krankheitsverständnis des*der Patient*in. Da dies jedoch deutlich schwerer messbar ist und QS klar definierte messbare Prozess- und Strukturkategorien benötigen, wurde sich letztlich auf die Anzahl durchgeführter strukturierter Patient*innenschulungen 2 Monate nach Diagnosestellung als messbare Prozesskategorie geeinigt.

Ebenfalls kritisch anzumerken ist, dass es zwar bereits einige Projekte u. a. der DRGh gibt, die sich aktuell mit der Entwicklung von strukturierten Patient*innenschulungen befassen [28], diese jedoch noch nicht ausreichend implementiert und etabliert sind (Tab. 8).

Nach erfolgten Änderungen stimmten 15 von 15 Mitgliedern für die finale Übersetzung.

### QS 8: Kurzfristiger Termin

Diskutiert wurde, ob die Übersetzung der englischen Originalversion der ASAS mit „Notfalltermine“ bestehen bleibt oder diese anders benannt werden sollte. In der Diskussion kam auf, dass es durchaus Situationen gibt, in denen ein*e Patient*in einen zeitnahen, sprich kurzfristigen, Termin braucht, ohne dass dies einen definierten medizinischen Notfall darstellt. Daher wurde sich mehrheitlich für die Umbenennung des Begriffes in „kurzfristiger Termin“ entschieden (Tab. 9).

Nach erfolgten Änderungen stimmten 15 von 15 Mitgliedern für die finale Übersetzung.

### QS 9: Jährliche Beurteilung

Während des virtuellen Treffens wurden die Komorbiditäten genauer definiert und die Erwerbssituation sowie die Lebensführung auf den Aspekt „Teilhabe“ subsumiert (Tab. 10).

Nach erfolgten Änderungen stimmten 15 von 15 Mitgliedern für die finale Übersetzung.

## Diskussion

Dies ist die erste Definition der DGRh von Qualitätsstandards in der Rheumatologie. Auf Grundlage der vor Kurzem publizierten internationalen ASAS-Empfehlungen für die Behandlung von axSpA-Patient*innen [29] und der soeben aktualisierten nationalen Leitlinie für das Management von Patient*innen mit axSpA [8] wurden 9 QS konsentiert, die messbar und geeignet sind, die Versorgungslage der Patient*innen zu verbessern. Die Themen entsprechen denen in der Originalpublikation: Überweisung, Zeit bis zum Termin bei Rheumatolog*innen, diagnostische Abklärung durch Rheumatolog*innen, Monitoring der Erkrankung, leitliniengerechte Therapieeskalation, nicht-pharmakologische Behandlung, Patient*innenschulung, Möglichkeit der kurzfristigen Terminvergabe und jährliche Beurteilung, hierbei geht es auch um Funktion, Mobilität und Komorbidität.

Bei den ersten 3 Punkten geht es v. a. um eine zeitnahe Diagnosestellung auf Expert*innenbasis – die wesentliche Voraussetzung für eine adäquate und rechtzeitige Therapie. Hierbei spielen die ASAS-Klassifikationskriterien von 2009 eine wichtige Rolle, die aber höchstens als grobe Orientierung dienen sollten [30]. Die Verfügbarkeit eines Magnetresonanztomographie(MRT)-Geräts und radiologische Expertise sind zusätzliche Qualitätsmerkmale für diese Domänen. Die Definition eines Therapieziels, in der Regel Remission, ist essenziell für ein zielgerichtetes Monitoring, wobei es im Verlauf dann v. a. darum geht, das Erreichen dieses Ziels durch standardisierte Assessments zu überprüfen. Im Vordergrund steht dabei die Messung der Krankheitsaktivität durch BASDAI bzw. ASDAS-CRP [31]. Wird das Therapieziel nicht erreicht, folgt eine leitliniengerechte Therapieeskalation [8].

Die ersten Symptome bei Patient*innen mit axSpA beginnen durchschnittlich im 26. Lebensjahr [1, 2]. Das bedeutet, dass es sich meist um Verläufe über mehrere Jahrzehnte handelt. Dabei ist auch von Bedeutung, dass die Morbidität und Mortalität für kardiovaskuläre Erkrankungen bei Patient*innen mit axSpA erhöht sind. Die Funktion der meisten Patient*innen mit axSpA verschlechtert sich im Verlauf [32], wobei mit zunehmender Krankheitsdauer die strukturellen röntgenologisch erfassbaren Wirbelsäulenveränderungen eine größere Rolle spielen als die entzündlichen, die in früheren Phasen der Erkrankung eine größere Bedeutung haben [33]. Neben den medikamentösen Einflussmöglichkeiten spielt die Physiotherapie im Management eine große Rolle für die Erhaltung von Funktion und Beweglichkeit [34, 35]. Dem wurde mit dem QS Nr. 6 Rechnung getragen. Hierzu, ebenso wie zum nächsten Thema, gehört auch die körperliche Aktivität, wozu es für Patient*innen mit muskuloskeletalen Erkrankungen aktuelle Empfehlungen der „European Alliance of Associations for Rheumatology“ (EULAR), früher „European League Against Rheumatism“ gibt [36].

Patient*innenschulung ist ein zunehmend wichtiges Thema in der Rheumatologie, denn es ist davon auszugehen, dass ein*e gut geschulte*r bzw. informierte*r Patient*in eine*n bessere*n Partner*in im Management auch im Sinne des „shared decision making“ darstellt. Die EULAR hat auch dazu Empfehlungen publiziert, und die DGRh hat sich in den letzten Jahren ebenfalls um eine Aktualisierung früherer Aktivitäten bemüht, erste Erfolge sind zu verzeichnen [37].

Auch bei Patient*innen mit axSpA gibt es Notfälle – wie etwa mögliche Medikamentennebenwirkungen, eine osteoporotische Wirbelkörperfraktur [38], eine akute Beteiligung des Atlantoaxialgelenks [39] oder neu aufgetretene kardiovaskuläre Komorbidität (z. B. arteriosklerotisch bedingte Ereignisse wie akuter Myokardinfarkt) [40–42]. Darüber hinaus gibt es aus Patient*innensicht noch zahlreiche andere „Notfälle“, die den Wunsch nach einem schnellen Termin begründen können. Dieser Situation wurde mit dem QS 8 Rechnung getragen.

Der letzte QS 9 beinhaltet die jährliche Überprüfung von Funktion, Mobilität, Indikationsstellung für Röntgenuntersuchungen oder andere bildgebende Verfahren im Verlauf, eine Knochendichtemessung, des Impfstatus, kardiovaskuläre Komorbidität (z. B. Blutdruckmessung, Cholesterin usw., wenn von dem*der Hausarzt*ärztin nicht bereits adressiert) und sozialmedizinische Aspekte (z. B. Antrag auf Rehabilitation, Antrag auf Schwerbehinderung, Rentenantrag usw.). Nicht nur hier kann man sich sicherlich Aufgaben für gut ausgebildete rheumatologische Fachassistent*innen vorstellen.

Zusammengefasst gibt es jetzt erstmalig in Deutschland evidenzbasierte messbare Qualitätsstandards, die eine gute Zielformulierung und -struktur für zukünftige Verbesserungen der Versorgung bieten.

## Fazit für die Praxis

- In Deutschland bestehen nach wie vor Versorgungslücken in der Versorgung von axSpA-Patient*innen.
- Erstmalig wurden nun evidenzbasierte Qualitätsstandards für die Behandlung von axSpA-Patient*innen in Deutschland entwickelt.
- Die Versorgung von axSpA-Patient*innen in Deutschland soll in Zukunft durch Verwendung von Qualitätsstandards messbar und das Outcome langfristig verbessert werden.

## Abkürzungen

AG: Arbeitsgruppe
ASAS: Assessment of SpondyloArthritis International Society
axSpA: Axiale SpA
DGRh: Deutsche Gesellschaft für Rheumatologie
DMARD: Disease-modifying anti-rheumatic drug
EULAR: European League Against Rheumatism
LL: Leitlinien
NICE: National Institute for Health and Care
NRS: Numerical rating skale
QS: Qualitätsstandards
SpA: Spondyloarthritiden
